# Changing epidemiology and antimicrobial resistance in *Vibrio cholerae*: AMR surveillance findings (2006–2016) from Nepal

**DOI:** 10.1186/s12879-019-4432-2

**Published:** 2019-09-11

**Authors:** Nisha Rijal, Jyoti Acharya, Shailaja Adhikari, Bishnu Psd Upadhaya, Geeta Shakya, Palpasa Kansakar, Piyush Rajbhandari

**Affiliations:** 1 National Public Health Laboratory, Kathmandu, Nepal; 2 Social Health Security Development Committee, Kathmandu, Nepal; 3 Patan Academy of Health Sciences, Kathmandu, Nepal

**Keywords:** *V.cholerae*, Antimicrobial resistance, Nepal, Antibiotics

## Abstract

**Background:**

In Nepal, cases of Cholera occur annually either as sporadic or as outbreaks claiming the lives of many in rural areas. The present study is a laboratory based surveillance which aims to analyze the changing epidemiology and antimicrobial susceptibility trend of *V. cholerae* strains isolated or referred to National Public Health Laboratory (NPHL) over a period of 11 years (2006–2016).

**Methods:**

Specimens of fresh stool /rectal swab either received at sentinel sites or NPHL were processed following standard microbiological techniques. Suspected colonies on selective medium were identified using routine biochemical tests and confirmed by serotyping. Antimicrobial susceptibility testing was performed following Kirby Baeur disc diffusion method.

**Results:**

Of the 836 confirmed isolates, 87% (728/836) were *V.cholerae* O1 Ogawa,12% (103/836) were *V.cholerae* O1 Inaba and only 6 isolates were *V.cholerae* O1 Hikojima. In 2006 all the *Vibrio* isolates were of Inaba serotype, followed by all 3 serotypes during 2007.During 2008–2014 only Ogawa serotype was isolated while few cases of Inaba again surfaced in 2015. Resistance to ampicillin decreased from 93% in 2006 to 18% by 2010 and again raised to 100% by 2016.Cotrimoxazole resistance remained at constant range (77–100%).Nalidixic acid resistance was 100% since 2006.Ciprofloxacin and tetracycline resistance emerged in 2007, reached a peak during 2010–2012 and declined to 0 by 2016.Susceptibility to Furazolidone has re-emerged.63.6% of the isolates were Multi drug resistant.

**Conclusion:**

With changing epidemiology and antibiogram of *V.cholerae* in Nepal, the present study reflects the importance of continuous monitoring, which could be used by policy makers and health professionals for better management of outbreaks. Decline in tetracycline and ciprofloxacin resistance along with emerging sensitivity to furazolidone shows that these drugs could make an effective comeback in future.

## Background

Cholera is an acute diarrheal illness caused by the bacterium *Vibrio cholerae* [1]. Until now, seven distinct pandemics of cholera have been recorded [2]. The earlier pandemics were caused by the classical biotype of *V. cholerae* O1, whereas the 7th ongoing pandemic is caused by serogroup O1 El Tor and has been the most extensive in geographical spread and duration [3]. Outbreak of epidemic cholera caused by a non-O1 strain of *V. cholerae* designated *V. cholerae* 0139 has also been reported from various parts of Bangladesh, India and other countries [4]. Globally, there are an estimated 3–5 million cholera cases and 100,000–120,000 deaths every year. Commonly, lack of prompt, proper treatment leads to shock within 6–12 h followed by death occurring between18 h and several days [5].

Over the past few years, *V. cholerae* O1 biotype El Tor causing Asiatic cholera has shown remarkable changes in its phenotypic and genetic characteristics [6]. The most recent development in the evolution of global cholera has been the emergence and spread of a new variant ET, or altered ET, in Bangladesh that carries *ctxB* of the classical (CL) biotype (*ctxB*CL) [7]. According to recent reports, altered ET strains have been spreading globally [8–15] causing more severe disease [16].

In Nepal, cholera is endemic. Every year, cases of cholera occur sporadically or as outbreaks. Diarrhea and gastroenteritis (including cholera and dysentery) comes in second position among top ten causes of inpatient morbidity and top ten reasons for OPD consultation. in Nepal (DoHS, 2017). It has also been included among one of the six outbreak potential diseases and are reported immediately through the Early Warning Reporting System (EWARS) to the National Epidemiology and Disease Control division (EDCD, 2017). In a decade, six episodes of laboratory confirmed cholera outbreak has been recorded by NPHL.

## Methods

**Fig. 1:**
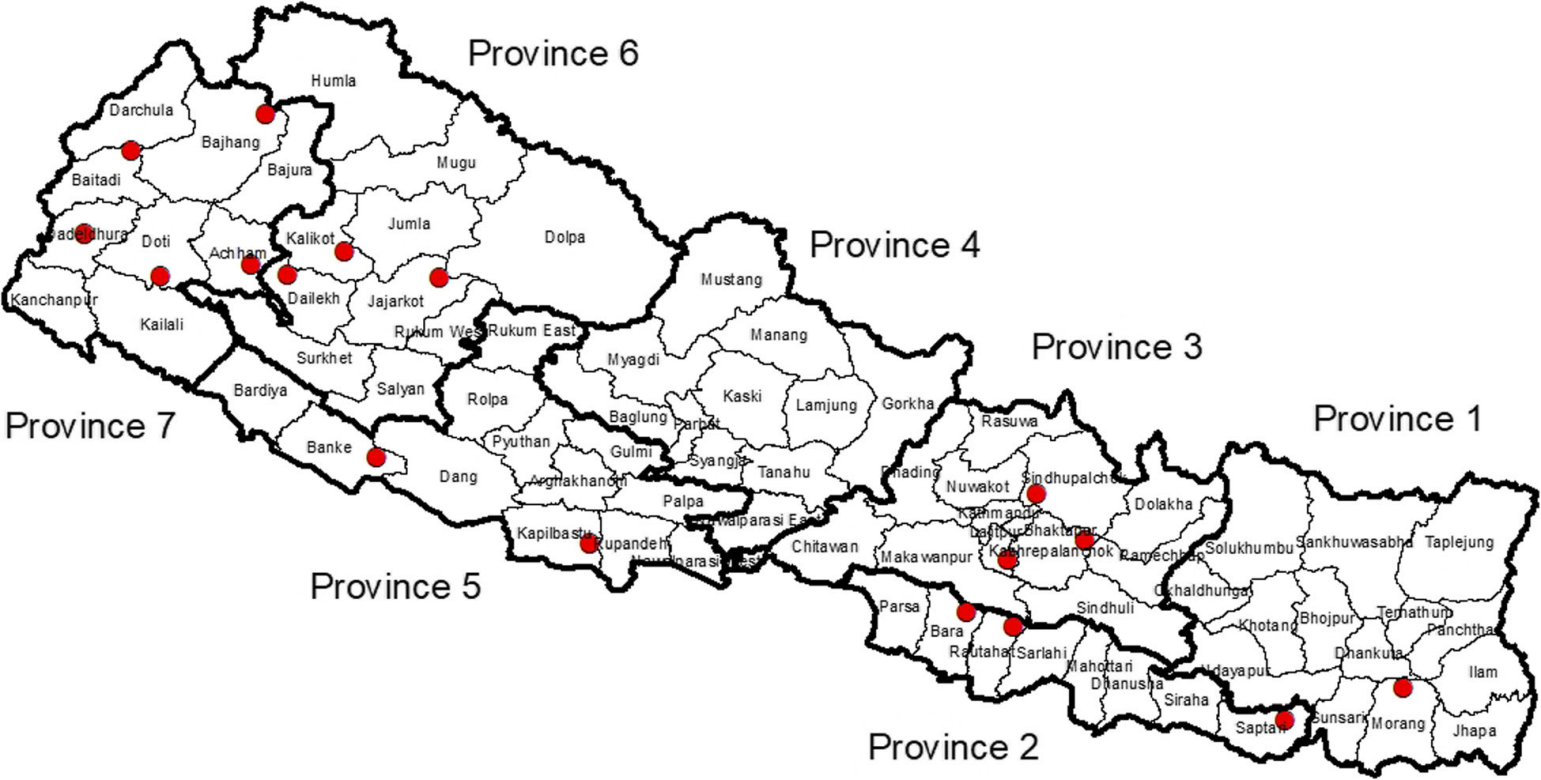
Map of Nepal showing cholera outbreaks in the past decade

A. Surveillance sites: There are 22 sentinel sites participating in laboratory based national antimicrobial resistance (AMR) surveillance. *V.cholerae* was isolated at 11 participating laboratories (Fig. 1)
B. Source of Vibrio:
  Suspected stool samples received at surveillance sites and samples from suspected outbreaks received at the reference laboratory were processed following standard microbiological procedures as mentioned below. Presumptive isolates from any source was confirmed, serotyped and stored at the reference laboratory.
C. Laboratory Methods:

1. Sample processing:
  Fresh stool/rectal swab samples were parallelly added to 5 ml of alkaline peptone water (APW) and streaked onto MacConkey agar and Thiosulphate Citrate Bile Salt Sucrose (TCBS) agar. Thus inoculated APW was incubated at 35–37 °C for 6–8 h for enrichment and the plates were incubated at 35–37 °C for 18–24 h.
  After enrichment, a loopful of APW was inoculated onto MacConkey agar and TCBS agar and then incubated at 35–37 °C for 18–24 h. Suspected colonies (i.e non-lactose fermenting on MacConkey agar or golden yellow colonies on TCBS) were identified by Gram stain, oxidase test, string test and inoculation on biochemical medium viz. Triple Sugar Iron agar, SIM medium, Urease agar, Citrate agar and MR-VP broth.
2. Serotyping: All isolates of *Vibrio* were confirmed serologically by slide agglutination using serogroup specific O1 polyvalent and O139 antisera and monospecific antisera for Ogawa & Inaba strains from Denka Seiken Company Limited, Tokyo, Japan. Strains showing visible agglutination were considered positive.
3. Biotyping: *Vibrio* isolates were biotyped using Vogues-proskaeur reaction and sensitivity to Polymyxin B (50 units) discs.

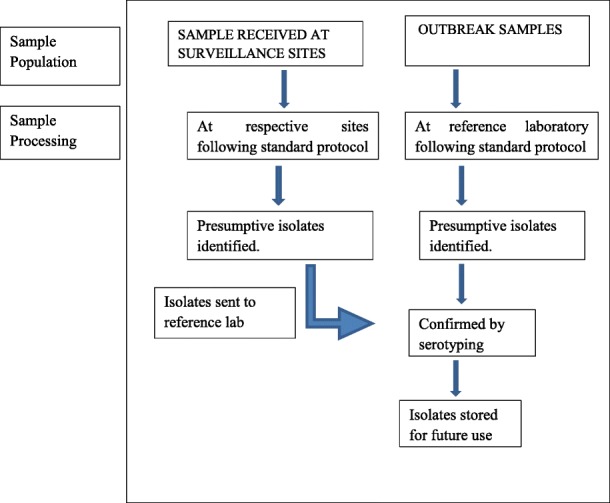



Flowchart for sample processing

D. Antibiotic susceptibility test was performed for all the identified *Vibrio* isolates by standard Kirby Bauer’s Disc Diffusion technique following updated CLSI guidelines [17]. The antibiotics tested were ampicillin (AMP-10mcg), cotrimoxazole (COT-25mcg), nalidixic acid (NA- 30mcg), ciprofloxacin (CIP-5mcg), tetracycline (T-30mcg), furazolidone (FR-100 units) and ceftriaxone (CRO-5 mcg). Strains of *Escherichia coli* ATCC 25922 were used as control.
E. Statistical methods: Data obtained were analyzed using SPSS software for windows version 18.Comparison of data with respect to Vibrio, sex, and age-groups were performed by Chi- square. *P* < 0.05 was considered to be statistically significant. Duplicate samples were counted as one during data entry.

## Results

**Fig. 2:**
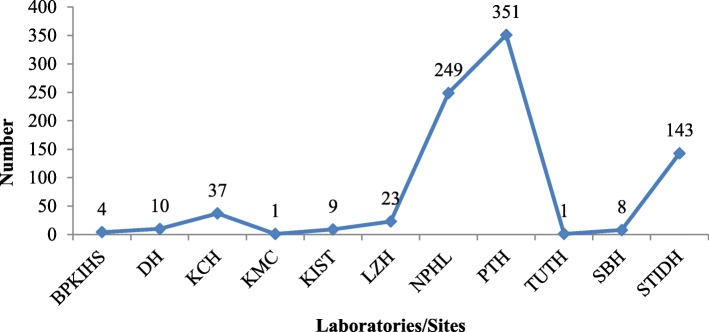
Laboratory wise distribution of cholera positive cases

Nepal reported 6 outbreaks in 18 districts (of 77), on 6 out of 7 provinces (Fig. 1), from 2006 to 2016. A total of 836 *V.cholerae* isolates were reported in a period of 11 years (2006–2016) from 11 participating laboratories including NPHL. The highest number was reported in 2007(Fig. 1). Among the participating laboratories, 42% were reported from Patan hospital, followed by NPHL and Sukraraj tropical and Infectious Disease Hospital (Fig. 2).

All the isolates verified at NPHL were of O1 serogroup and Eltor biotype. Of the total 87% (724) were *V.cholerae* O1 Ogawa, 12% (103) were *V.cholerae* O1 Inaba and only 1% [6] isolates were of *V.cholerae* O1 Hikojima serotype. However the prevalent serotype kept changing over the years. In 2006, *V. cholerae* O1 Inaba was predominant, by 2007 all serotypes *V. cholerae* O1 Eltor Ogawa, Inaba & Hikojima were observed. During 2008–2016, *V. cholerae* O1 Ogawa predominated again with one case each of *V. cholerae* O1 Inaba in recent years (Table 1).

**Table 1:** Biotype, Serogroup and serotype distribution of *V. cholerae* in Nepal (2006–2016)

Year	Total isolates	Biotype	Serogroup	Serotypes		
				Ogawa	Inaba	Hikojima
2006	32	ElTor	O1	0	28	4
2007 & 2008	352	ElTor	O1	278	73	2
2009–2014	205	ElTor	O1	205	0	0
2015 & 2016	247	ElTor	O1	245	2	0

**Fig. 3:**
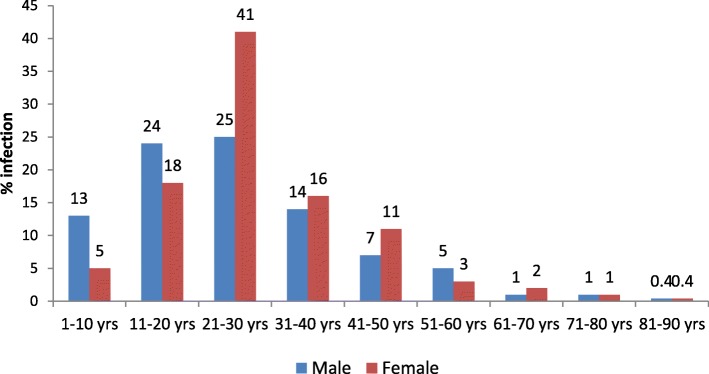
Age and gender wise distribution of positive cases

Cholera cases peaked in monsoon season (June to august). Overall, infection was higher in males as compared to females but no significant difference was observed. Among both the gender, infection was predominant in 21–30 years age group (Fig. 3).

**Fig. 4:**
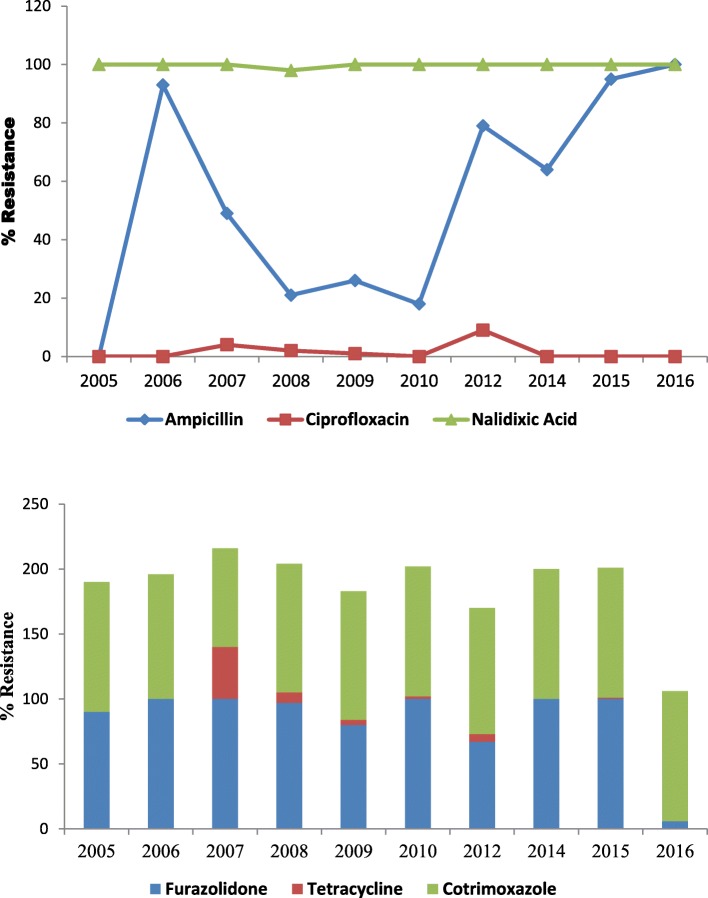
Antibiogram of *V.cholerae* isolates (2006–2016)

Rapid fluctuation in antimicrobial susceptibility trend among *Vibrio cholerae* was observed over the years and is demonstrated in Fig. 4.Resistance to ampicillin decreased from 93% in 2006 to 18% by 2010 and again rose to 100% by 2016. Cotrimoxazole resistance fluctuated between 76 and 100%. All the isolates were sensitive to ciprofloxacin till 2006; resistance emerged in 2007, peaked during 2012 and declined to 0 by 2016. After several years in which *V. cholerae* was uniformly susceptible to tetracycline, a sudden upsurge in tetracycline resistance was noted, from 0% in 2006 to 40% in 2007 before decreasing to 8% in 2009. A significant decline in furazolidone resistant isolates was observed in this study period (100% in 2006 to 5% in 2016). All the isolates were sensitive to ceftriaxone; however since 2013, isolates showing partial susceptibility has also been reported. In 2016, one isolate was resistant to ceftriaxone.

Of the total, 61.9% isolates (518/836) were Multidrug resistant. Of them, 4.1% (21/518) were resistant to five classes of antibiotics, 34.5% (179/518) were resistant to four classes and 61% (357/532) were resistant to three classes of antibiotics tested. The most common resistotype was isolates exhibiting simultaneous resistance to ampicillin, cotrimoxazole and quinolones (Table 2).

**Table 2:** Multidrug resistant phenotypes of *V.cholerae*

Property	MDR phenotype	Number of isolates exhibiting resistance
Resistance to 5 classes of antibiotics		
	Amp/Cot/Cip or NA/T/FR	21
Resistance to 4 classes of antibiotics		
	Amp/Cip or NA/Cot/FR	166
	Cot/NA/T/FR	10
	Other resistotypes	3
Resistance to 3 classes of antibiotics		
	Cot/Cip or NA/FR	98
	Amp/Cip or NA/Cot	194
	Amp/Cip or NA/FR	17
	Other resistotypes	9
Total		518

*Amp* Ampicillin, *CIP* Ciprofloxacin, *COT* Cotrimoxazole (trimethoprim sulfomethoxazole), *NA* Nalidixic acid,*T* tetracycline, *FR* Furazolidone, *CTR* Ceftriaxone

## Discussion

Cholera, its sudden and explosive onset in the form of an outbreak or epidemic, coupled with high mortality and morbidity rates, still persists as a major health problem in Nepal. The present article gives a review of the cholera scenario based on biotype, serotype and antimicrobial susceptibility in our area from 2006 to 2016.

All the *V.cholerae* isolated over 11 years was of O1 serogroup, no isolate of non-O1 serogroup has been reported from Nepal so far. However, outbreaks due to non-O1 serogroups have been reported from India and Bangladesh [18–21]. In our study, all the *V.cholerae* isolates received and verified at NPHL were of El Tor biotype, which is in accordance to other studies [22–26] but contrary to study made by Rojina et al., in 2012, which reports 22 *V.cholerae* isolated at Sukraraj tropical and Infectious disease hospital to be of Classical Biotype [27]. Since, the hospital was included in AMR surveillance since 2013 only, the isolates were reported but not verified at NPHL.

Over a period of 11 years, interesting changes in prevalent serotype of *V.cholerae* was observed. Studies made before 2005, revealed Ogawa as the predominant serotype whereas during 2006, Inaba predominated. By 2007, all three serotypes were found. After nearly 9 years, by 2015 and 2016, one case of Inaba was seen, which may be an indication that gradually there may again be a conversion. Periodic shifts in the incidence of Ogawa and Inaba serotypes in a given area is a common phenomenon and is thought to be a consequence of genetic reversal that occurs both in vitro and in vivo [28, 29]. Such observation has been reported from various national and international studies [18, 30–32].

Up to now, all the reported outbreak and sporadic cases were mostly concentrated in Central and Far-western part of the country; however, few cases were also reported from eastern part. The hill districts of the mid and far western development regions are particularly affected due to its remoteness, poor and difficult accessibility, inadequate public health facilities and poor water and sanitation conditions. In the central region the supply of safe drinking water is inadequate due to increase in urban population density due to which people are forced to drink water from any available source with or without prior treatment. Other factors like floods, landslides, open defecation practices, breakdown in sanitation and in the supply of safe water also contribute to frequent cases of cholera [33].

Overall infection was higher in males, of adult age group (21–30 yrs). Study conducted by Uthappa et al. also supports 12% attack rate in males, but higher incidence in children of 6–14 years age group. Studies made by Bhandari et al. and Gupta et al. in Nepal have reported 33.3% in 15–44 years age group and 48.8% in 20–39 years age group, similar to our study [33, 34].

Resistance patterns in *V. cholerae* are known to fluctuate rapidly because it cannot stably carry plasmids that confer resistance and because the organism naturally resides in aquatic environments devoid of selective pressure from antibiotics [35, 36]. Our study has also shown dramatic fluctuations in resistance to routinely used antibiotics. High resistance against cotrimoxazole, nalidixic acid and furazolidone (90–100%) was evident since 2006. Such high resistances to cotrimoxazole and nalidixic acid have also been reported from Mozambique [37, 38], Zambia [39], India [40–42] and Haiti [43]. Along the years, ampicillin resistance fluctuated. Some studies have shown 100% resistance [39, 44] while some has shown increasing resistance [45].

The reason behind the sudden upsurge and subsequent decline in ciprofloxacin and tetracycline resistance is unknown; however, the decline in tetracycline resistance has also been reported from other areas [40, 42, 43]. Studies made by Karlsson et al. [43] and Mala et al. [46] have reported susceptibility to tetracycline similar to our current scenario. Similarly, studies conducted at Mozambique [39], Bangladesh [47] also shows susceptibility to ciprofloxacin, whereas some other studies show resistance fluctuating between 2 and 39% [41].

One of the important finding in our study was re-emergence of sensitivity to furazolidone. During our study period, furazolidone resistance remained 80–100% (except in 2012), and abruptly in 2016, a significant decline in resistance to 6% was noted. Klontz et al., in his study, have stated that they stopped consistently testing for resistance to furazolidone due to high resistance levels [47]. Most of the previous national and international studies have reported 100% isolates resistant to furazolidone [23, 48]. Some other studies have shown increasing resistance from 21% in 2008 to 95% in 2010 [45]. Recently, only 1 *Vibrio* was resistant to ceftriaxone, which may be an indication that, in near future, ceftriaxone resistance may also emerge. So far, no study has reported ceftriaxone resistance in *Vibrio*.

In our context, the origin of MDR *Vibrio* strain is not documented, however, studies made before 2006, have reported MDR (defined it as simultaneous resistance to 2 different classes of antibiotics) cholera cases [25]. With the current definition of MDR (isolates resistant to one or more of 3 or more different classes, our result shows that 94% isolates were MDR in 2006 reaching to 100% by 2016 which is in concordance to results obtained by Shrestha et al. [49]. Since, cholera was well prevalent in Nepal before this period, MDR strains may have emerged presumably due to heavy and widespread use of antibiotics for therapy.

## Conclusion

Since the emergence of antibiotic resistance may significantly influence patient care, continuous monitoring of epidemic strains is crucial. Molecular studies emphasizing on the Although few isolates were further characterized for ctxB gene, MLVA typing, the genes and mechanisms responsible for antibiotic resistance still remains an area for further exploration.

## Data Availability

The datasets used and analyzed during the current study are available from the corresponding author on reasonable request.

## Abbreviations

Amp: Ampicillin
AMR: Antimicrobial Resistance
APW: Alkaline Peptone Water
Cot: Cotrimoxazole
CRO: Ceftriaxone
Ctx-B: Cholera enterotoxin subunit B
EDCD: Epidemiology and Disease Control Division
EWARS: Early Warning Reporting System
FR: Furazolidone
MDR: Multidrug Resistant
MLVA: Multiple-Locus Variable number tandem repeat analysis
MR-VP: Methyl Red-Vogues Proskaeur
NA: Nalidixic Acid
NPHL: National Public Health Laboratory
SIM: Sulfur Indole Motility
T: Tetracycline
TCBS: Thiosulphate Citrate Bile Salt Sucrose
TSI: Triple Sugar iron
